# Supplementary material for: Exocrine and Endocrine Insufficiency in Autoimmune Pancreatitis: A Matter of Treatment or Time?
Source: J Clin Med. 2022 Jun 28;11(13):3724. doi: 10.3390/jcm11133724 (PMC9267378; doi:10.3390/jcm11133724)
Supplement: Supplementary file 1 [file jcm-11-03724-s001.zip › jcm-1773178-supplementary.pdf]

Supplement

Table S1: Characteristics of patients with AIP and their association with the presence of PEI at baseline.

	Total<br>N (% col)	PEI at diagnosis<br>N (% row)	P-value Fisher's Exact test	Total	PEI at last follow-up	P-value Fisher's Exact test
Total	44 (100.)	32 (72.7)		52 (100.0)	33 (63.5)	
Sex						
Male	31 (70.5)	24 (77.4)		36 (69.2)	25 (69.4)	
Female	13 (29.5)	8 (61.5)	0.30	16 (30.8)	8 (50.0)	0.22
Age at AIP diagnosis						
0-39	8 (18.2)	3 (37.5)		10 (19.2)	3 (30.0)	
40-49	3 (6.8)	3 (100.)		3 (5.8)	2 (66.7)	
50-59	6 (13.6)	4 (66.7)		7 (13.5)	4 (57.1)	
60-69	13 (29.5)	11 (84.6)		19 (36.5)	15 (78.9)	
70+	13 (29.5)	10 (76.9)	0.16	13 (25.0)	9 (69.2)	0.12
BMI at diagnosis						
Unknown	1 (2.3)	0 (0.0)		2 (3.8)	0 (0.0)	
<25 kg/m2	22 (50.0)	14 (63.7)		25 (48.1)	18 (72.0)	
≥25 kg/m2	21 (47.7)	18 (85.7)	0.16	25 (48.1)	15 (60.0)	0.55
Collar profession						
Unknown	8 (18.2)	5 (62.5)		9 (17.3)	8 (88.9)	
White	26 (59.1)	18 (69.2)		30 (57.7)	17 (56.7)	
Blue	10 (22.7)	9 (90.0)	0.39	13 (25.0)	8 (61.5)	1.00
Alcohol						
Unknown	3 (6.8)	2 (66.7)		3 (5.8)	2 (66.7)	
No alcohol	40 (90.9)	29 (72.5)		48 (92.3)	30 (62.5)	
>5U/day	1 (2.3)	1 (100.)	1.00	1 (1.9)	1 (100.)	1.00
Smoking						
Unknown	4 (9.1)	3 (75.0)		3 (5.8)	2 (66.7)	
Never	23 (52.3)	16 (69.6)		28 (53.8)	22 (78.6)	
Ever	17 (38.6)	13 (76.5)	0.73	21 (40.4)	9 (42.9)	**0.01**
Abdominal pain						
No	22 (50.0)	16 (72.7)		27 (51.9)	18 (66.7)	
Yes	22 (50.0)	16 (72.7)	1.00	25 (48.1)	15 (60.0)	0.77
Weight loss						
No	31 (70.5)	21 (67.7)		36 (69.2)	22 (61.1)	
Yes	13 (29.5)	11 (84.6)	0.46	16 (30.8)	11 (68.8)	0.76
Obstructive jaundice						
No	20 (45.5)	11 (55.0)		22 (42.3)	11 (50.0)	
Yes	24 (54.5)	21 (87.5)	**0.02**	30 (57.7)	22 (73.3)	0.14
Acute pancreatitis						
No	34 (77.3)	27 (79.4)		42 (80.8)	28 (66.7)	
Yes	10 (22.7)	5 (50.0)	0.11	10 (19.2)	5 (50.0)	0.47
Asymptomatic						
No	38 (86.4)	28 (73.7)		45 (86.5)	30 (66.7)	
Yes	6 (13.6)	4 (66.7)	0.66	7 (13.5)	3 (42.9)	0.40

Diabetes at diagnosis						
No	31 (70.5)	20 (64.5)		34 (65.4)	21 (61.8)	
Yes	13 (29.5)	12 (92.3)	0.07	18 (34.6)	12 (66.7)	0.77
IgG4 value at diagnosis						
Unknown	2 (4.5)	2 (100.)		1 (1.9)	1 (100.)	
Negative	20 (45.5)	13 (65.0)		25 (48.1)	14 (56.0)	
Positive	12 (27.3)	9 (75.0)		14 (26.9)	9 (64.3)	
2x normal value	10 (22.7)	8 (80.0)	0.75	12 (23.1)	9 (75.0)	0.56
Focal enlargement						
Unknown	2 (4.5)	1 (50.0)		1 (1.9)	1 (100.)	
No	32 (72.7)	24 (75.0)		34 (65.4)	22 (64.7)	
Yes	10 (22.7)	7 (70.0)	1.00	17 (32.7)	10 (58.8)	0.76
Diffuse						
Unknown	2 (4.5)	1 (50.0)		1 (1.9)	1 (100.)	
No	14 (31.8)	9 (64.3)		21 (40.4)	11 (52.4)	
Yes	28 (63.6)	22 (78.6)	0.46	30 (57.7)	21 (70.0)	0.25
Steroids						
No	9 (20.5)	6 (66.7)		8 (15.4)	5 (62.5)	
Yes	35 (79.5)	26 (74.3)	0.69	44 (84.6)	28 (63.6)	1.00
Azathioprine						
No	37 (84.1)	28 (75.7)		44 (84.6)	27 (61.4)	
Yes	7 (15.9)	4 (57.1)	0.37	8 (15.4)	6 (75.0)	0.69
Rituximab						
No	34 (77.3)	24 (70.6)		42 (80.8)	24 (57.1)	
Yes	10 (22.7)	8 (80.0)	0.70	10 (19.2)	9 (90.0)	0.07
Biologics						
No	42 (95.5)	31 (73.8)		51 (98.1)	33 (64.7)	
Yes	2 (4.5)	1 (50.0)	0.48	1 (1.9)	0 (0.0)	0.37
Stent						
No	26 (59.1)	17 (65.4)		29 (55.8)	14 (48.3)	
Yes	18 (40.9)	15 (83.3)	0.30	23 (44.2)	19 (82.6)	**0.02**
Maintenance treatment						
No	25 (56.8)	17 (68.0)		31 (60.4)	16 (51.6)	
Yes	19 (43.2)	15 (78.9)	0.50	21 (39.6)	17 (81.0)	**0.04**
OOI						
No	3 (6.8)	0 (0.0)		3 (5.7)	0 (0.0)	
Yes	41 (93,2)	32 (78.0)	**0.01**	59 (94.2)	33 (67.3)	**0.04**

AIP=autoimmune pancreatitis; PEI=pancreatic exocrine insufficiency; DM=diabetes mellitus; fup=follow-up; BMI=body mass index; OOI=other organ involvement

Table S2. Characteristics of patients with AIP and their association with the presence or development of diabetes mellitus.

	Total N (% col)	DM at diagnosis N (% row)	P-value Fisher's Exact test	DM During fup Change	P-value Log-rank test New DM	P-value Log-rank test Any time
	58 (100.0)	19 (32.8)		+8		
Sex						
Male	40 (69.0)	14 (35.0)		+6		
Female	18 (31.0)	5 (27.8)	0.77	+2	0.65	0.48
Age at AIP diagnosis						
0-39	11 (19.0)	1 (9.1)		+1		
40-49	3 (5.2)	1 (33.3)		+1		
50-59	7 (12.1)	2 (28.6)		+2		
60-69	22 (37.9)	11 (50.0)		+3		
70+	14 (24.1)	4 (28.6)	0.14	+1	0.70	0.20
BMI at diagnosis						
Unknown	2 (3.4)	1 (50.0)		=		
<25 kg/m2	29 (50.0)	5 (17.2)		+4		
≥25 kg/m2	27 (46.6)	13 (48.1)	**0.04**	+4	0.35	**0.01**
Collar profession						
Unknown	11 (19.0)	4 (36.4)		+2		
White	32 (55.2)	6 (18.8)		+3		
Blue	15 (25.9)	9 (60.0)	**0.04**	+3	0.14	**0.002**
Alcohol						
Unknown	5 (8.6)	3 ()		+2		
No alcohol	52 (89.7)	15 (28.8)		+6		
>5U/day	1 (1.7)	1 (100.)	0.30	=	-	0.13
Smoking						
Unknown	5 (8.6)	3 (60.0)		=		
Never	31 (53.4)	6 (19.4)		+5		
Ever	22 (37.9)	10 (45.5)	0.07	+3	0.46	**0.04**
Abdominal pain						
No	32 (55.2)	13 (40.6)		+3		
Yes	26 (44.8)	6 (23.1)	0.27	+5	0.53	0.49
Weight loss						
No	38 (65.5)	10 (26.3)		+4		
Yes	20 (34.5)	9 (45.0)	0.14	+4	0.14	**0.04**
Obstructive jaundice						
No	25 (43.1)	3 (12.0)		+2		
Yes	33 (56.9)	16 (48.5)	**0.009**	+6	0.06	**0.0006**
Acute pancreatitis						
No	47 (81.0)	16 (34.0)		+8		
Yes	11 (19.0)	3 (27.3)	1.00	=	0.09	0.14
Asymptomatic						
No	51 (87.9)	19 (37.3)		+7		
Yes	7 (12.1)	0 (0.0)	0.09	+1	0.92	0.11

Elastase-1 at diagnosis						
Unknown	14 (24.1)	6 (42.9)		+2		
≥ 200µg/g	12 (20.7)	1 (8.3)		+1		
< 200µg/g (PEI)	32 (55.2)	12 (37.5)	0.13	+5	0.17	**0.02**
IgG4 value at diagnosis						
Unknown	2 (3.4)	0 (0.0)		=		
Negative	27 (46.6)	8 (29.6)		+3		
Positive	17 (29.3)	8 (47.1)		+2		
2x normal value	12 (20.7)	3 (25.0)	0.32	+3	0.40	0.49
Focal enlargement						
Unknown	2 (3.4)	0 (0.0)		=		
No	38 (65.5)	15 (39.5)		+6		
Yes	18 (31.0)	4 (22.2)	0.36	+2	0.48	0.15
Diffuse						
Unknown	2 (3.4)	0 (0.0)		=		
No	23 (39.7)	4 (17.4)		+3		
Yes	33 (56.9)	15 (45.5)	0.08	+5	0.33	**0.02**
Steroids						
No	10 (17.2)	3 (30.0)		=		
Yes	48 (82.8)	16 (33.3)	1.00	+8	0.14	0.26
Azathioprine						
No	49 (84.5)	16 (32.7)		+5		
Yes	9 (15.5)	3 (33.3)	1.00	+3	0.18	0.36
Rituximab						
No	48 (82.8)	16 (33.3)		+5		
Yes	10 (17.2)	3 (30.0)	1.00	+3	0.18	0.47
Biologics						
No	56 (96.6)	19 (33.9)		+8		
Yes	2 (3.4)	0 (0.0)	1.00	=	0.59	0.25
Stent						
No	32 (55.2)	6 (18.8)		+2		
Yes	26 (44.8)	13 (50.0)	**0.04**	+6	**0.005**	**0.0002**
Maintenance treatment						
No	36 (62.1)	12 (33.3)		+3		
Yes	22 (37.9)	7 (31.8)	1.00	+5	0.11	0.37
OOI						
No	3 (5.2)	0 (0.0)		=		
Yes	55 (94.8)	19 (34.6)	0.54	+8	0.33	0.12

AIP=autoimmune pancreatitis; PEI=pancreatic exocrine insufficiency; DM=diabetes mellitus; fup=follow-up; BMI=body mass index
